# Radiation therapists' perspectives on participating in research

**DOI:** 10.1002/jmrs.237

**Published:** 2017-07-21

**Authors:** Georgia K. B. Halkett, Melissa Berg, Martin A. Ebert, David Cutt, Michael Davis, Desley Hegney, Michael House, Michelle Judson, Rachel Kearvell, Michele Krawiec, Leanne Lester, Sharon Maresse, Peter McLoone, Jan McKay

**Affiliations:** ^1^ School of Nursing, Midwifery and Paramedicine Faculty of Health Sciences Curtin University Perth Western Australia Australia; ^2^ Radiation Oncology Sir Charles Gairdner Hospital Nedlands Western Australia Australia; ^3^ School of Physics University of Western Australia Crawley Western Australia Australia; ^4^ Genesis Cancer Care Perth Western Australia Australia; ^5^ Research Division Central Queensland University Rockhampton Queensland Australia; ^6^ School of Nursing and Midwifery The University of Southern Queensland Toowoomba Queensland Australia; ^7^ School of Nursing The University of Adelaide Adelaide South Australia Australia; ^8^ Health Promotion Evaluation Unit School of Sport Science, Exercise and Health University of Western Australia Crawley Western Australia Australia; ^9^ Discipline of Medical Radiation Science Faculty of Science and Engineering Curtin University Perth Western Australia Australia

**Keywords:** barriers, career, radiation therapist, research, tasks

## Abstract

**Introduction:**

The objectives of this research were to: (1) determine the extent of Australian radiation therapists (RTs) research participation; (2) evaluate the impact of research involvement on career perceptions (3) explore which research topics require investigation and (4) identify benefits and barriers to research participation.

**Methods:**

This study used mixed methods to collect qualitative and quantitative data using an online survey from a larger workforce study of RTs and radiation oncology medical physicists. Participants practising in Australia completed questions about their research involvement. Chi‐square tests and logistic regression were used to analyse quantitative data and content analysis was used to explore qualitative data.

**Results:**

Two hundred and ninety‐six RTs answered the research questions. Forty‐six percent had been involved in research. Of these, 91% had been involved in departmental, 28% in national, 14% in international and 29% in informal or self‐directed research studies. Eleven RTs (8%) had received funding as a chief/principal investigator. Involvement in research was associated with a desire to make a career change. However, it also appeared to be associated with greater satisfaction with career progression and staying in the career. Respondents identified a range of potential research topics, benefits of participating in research and barriers which included lack of time, support and cost.

**Conclusion:**

Almost half of the RT participants identified that they were participating in research. Our data suggest that continued involvement in research, and opportunities to participate, improve RT job satisfaction. RTs' research activities are likely to be extended through provision of additional time and support.

## Introduction

Radiation therapy is an evolving health field which requires extreme precision and accuracy to design and deliver high‐dose radiation treatment to tumours while minimising radiation to the surrounding organs. Research plays an essential role in informing evidence‐based practice to ensure high‐quality treatment and care is provided to patients.^1^ In the past 10 years in Australia, the involvement of radiation therapists (RTs) in research has increased with more RTs participating in clinical trials, projects that are relevant to changing radiation therapy practice, and enrolling in Higher Degrees by Research.^2^ It is not known; however, how many RTs are currently involved in designing and leading research projects or whether Australian RTs have the opportunity to participate in research and develop their research skills.

Very few RTs were involved in research in Australia in the early 2000s and at that time RTs lacked confidence in their research abilities, had limited time for research involvement and did not have the necessary support to participate.^3^ The following barriers to research involvement were identified in Canada: workplace culture, time, support, education and training, and personal barriers.^4^ Higgins et al.^5^ identified enablers to conducting research which included having a network of experienced researchers and access to departmental hardware and software resources.

In 2005, Agustin et al.^6^ surveyed 78 RTs to find that insufficient time was the main barrier to clinical trials research participation. Other enabling factors for research participation included promoting research in job descriptions, recognising research productivity in career advancement and more opportunities to actively participate in clinical trials. Agustin et al.^6^also highlighted RTs need for support and mentorship during research participation.

Wright, Hilder and Schneider‐Kolsky surveyed 36 Australian clinical centres in 2007 to gain insight into the status of Australian radiation therapy research.^2^ They found 36% of clinical centres had research RTs. The role of research RTs included facilitating and conducting research, collaboration and quality assurance. RTs were involved in a range of research studies and in most centres (78%), RTs initiated their own research studies. Sixty‐seven percent of clinical centres were involved in national, international, pharmaceutical and equipment clinical trials sponsored by industry and 39% of centres participated in multicentre studies. Funding for research was obtained from: National Health and Medical Research Council (NHMRC), Australian Institute of Radiography (now known as Australian Society of Medical Imaging and Radiation Therapy (ASMIRT)), State Cancer Councils, Cancer Institute of NSW, Victorian Cancer Agency, other medical research institutes and hospital funding. This article also found that small numbers of RTs (*n* = 32) in the participating clinical centres were completing or had completed Higher Degrees by research and 52 peer‐reviewed publications had been published in the previous 5 years.^2^

Previous studies have also determined which research topics are of interest and important to RTs practising in the clinical environment. In the late 2000s, a Delphi study was conducted to determine research priorities of Australian RTs demonstrating that a wide range of research is required in radiation therapy in the areas of technology, patient care and focusing on the workforce.^7^, ^8^, ^9^ Similar research priorities for RTs were also identified using a Delphi study in Norway.^10^

As the importance of RTs being involved in research is now established as part of the profession, it is important that we determine how actively RTs are involved in research and explore current barriers and benefits of research participation. The objectives of this research were to: (1) determine the current extent of RTs research participation; (2) evaluate the impact of involvement in research projects on career perceptions (3) explore RTs perspectives on which research topics require investigation and (4) identify perceived benefits and barriers to research participation.

## Methods

This study collected qualitative and quantitative data using an online survey from a larger workforce survey conducted with RTs and radiation oncology medical physicists (ROMPs).^11^ We have also reported ROMPs' perspectives on undertaking research elsewhere.^12^

Ethical approval was obtained from by Curtin University's Human Research Ethics Committee (RD‐25‐13).

### Recruitment

An email survey invitation was distributed to radiation therapists via the Australian Institute of Radiography (now known as Australian Society of Medical Imaging as Radiation Therapy (ASMIRT)), via Chief RTs email list in Australian oncology treatment centres (*n* = 70), the Medical Radiation Practice Board of Australia, Genesis Cancer Care National Network, the Australasian Radiation Therapy Clinical Educator network email list, and also using print media at conferences. Authors also contacted colleagues and utilised distribution lists in other countries to assist in distributing the surveys internationally. Facebook posts on relevant professional bodies' pages were also used. Indirect distribution of the surveys prevented response rates from being calculated.

### Instrument

The Workforce Sustainability in Radiation Oncology (WSRO) instrument was developed based on previous work exploring workforce issues in nursing and other professions in Australia.^13^, ^14^, ^15^ RTs completed questions relating to demographics, qualifications, current employment, career, future intentions, research, professional development, radiation oncology practice exit and previous or current employment in Australia. An expert panel (*n* = 8) including RTs, ROMPs, and researchers assessed the survey for clarity, content validity, internal consistency and uniqueness of each question.^16^, ^17^ Items with less than the minimum criterion for agreement were adapted or deleted based on feedback received and in consultation with the expert panel members.^18^ Responses relating to participants perspectives on research and research opportunities (11 possible items: 3 binary response items; 1 4‐point and 5‐point Likert type scales; 1 multiple response item; and 5 open ended) are reported separately here. All participants practising in Australia were asked to complete the questions on research.

### Procedure

After viewing the information sheet and consent form, RT participants were invited to complete the survey hosted on Qualtrics^®^ as an open link accessed via the project website. Participants currently working in Australia could enter a lottery for a chance to win one of thirteen AUD$50 vouchers. Data collection occurred from 30th September 2013 to the 2nd May 2014.

### Data analysis

SPSS Version 21 was used to analyse the data. Chi‐square tests were used to test for significant differences between groups with respect to personal demographics (age, gender, country of birth, relationship status (in a relationship vs. not), and having dependants), employment (qualifications (bachelor/masters vs. not), country of qualification, additional qualification, years of experience, work location, full‐time or part‐time, overtime hours, service provider type, completed a competency‐based assessment, moved to Australia for work or study, had a break in practice, and completion of a professional development year), satisfaction with career progression and advancement opportunities, intention to leave the profession (leave vs. unsure/not leave), or change career (change vs. unsure/no intention). Due to low numbers in some cells, 5‐point scales were collapsed to 3‐point scales (e.g. strongly agree collapsed with agree, neither agree nor disagree, strongly disagree collapsed with disagree). When overall probability in the Chi‐square test was *P* < 0.05, inter‐group comparisons were determined by *z*‐tests (adjusted Bonferroni method) to determine which cells in the chi‐square were significantly different and only those were reported. Although this was a national survey, a secondary focus was workforce sustainability in WA and therefore WA participants were compared to participants nationally. Multiple regressions were conducted using binary logistic regression (backwards elimination conditional method) using a *P* = 0.10 value for exclusion to investigate the relative impact of demographic variables on previous research experience; firstly with all personal demographic predictors (described above) and then again with significant predictors added to a model of employment predictors (also described above). All variables used for regression analyses were converted to binary form and each analysis started with the full model of variables, before insignificant predictors were removed to produce the most parsimonious model. Kendall's tau was used to correlate the total number of projects (excluding informal/self‐directed projects) with the type of research projects (departmental = 1, national = 2, and international = 3) RTs had been involved in.

Text from each open ended question was analysed using a summative content analysis whereby categories and subcategories were derived directly from participants' responses and counted.^19^, ^20^ Three stages were used for coding: first, a relevant code was applied to responses by three authors (GH, MB, MJ); second, the codes were grouped into categories/topics; and finally, responses in these categories/topics and subcategories/sub‐topics were counted. Category counts were ranked from highest to lowest and tabulated.

## Results

### Demographics

For the overall workforce survey we received responses from 342 RTs, of which 322 (94%) were currently practising. Two hundred and ninety‐six (87%) RTs answered the survey section about research. Forty‐six percent of RTs (*n* = 136, 95% CI (40, 52)) who responded to the research questions responded yes to the question “are you currently, or have you previously, been involved in research project/s?”. Participants' demographics are shown in Table 1.

**Table 1:** RT participant's demographic characteristics

*N* = 296		
	Mean	Standard deviation
Age (years) (range 20–66)	37.4	11.03
	*n*	%
Age		
≤30	101	34.4
>30	193	65.6
Gender		
Male	68	23.1
Female	227	76.9
Country of birth		
Australia	214	72.3
Overseas	82	27.7
Moved to Australia for study or work		
Yes	39	16.0
No	205	84.0
Relationship status		
Single	57	19.3
Married/de facto	210	70.9
In a relationship (not cohabiting)	22	7.4
Widowed	3	1.0
Divorced/separated	4	1.4
Dependents		
Yes	62	20.9
No	234	79.1
Entry/base qualification		
Certificate	8	2.7
Diploma	62	20.9
Bachelor	191	64.5
Master degree	28	9.5
Other	7	2.4
Country of entry/base qualification		
Australia	253	85.5
International	43	14.5
Completed a competency‐based assessment		
Yes	64	21.8
No	230	78.2
Additional qualifications		
Yes	78	26.6
No	215	73.4

Twenty percent (*n* = 60, 95% CI (16, 25)) of participating RTs had or were progressing towards a post‐graduate qualification; for five RTs this was identified as a research focussed qualification. Participants' higher degree studies were: Master by coursework (*n* = 9 enrolled, *n* = 51 completed), Master of Philosophy (*n* = 1 enrolled), and Doctorate of Philosophy (*n* = 2 enrolled, *n* = 2 completed) (Table 2).

**Table 2:** RT participant's employment demographic characteristics

*N* = 296	*n*	%
Years' experience		
≤10 years	144	48.6
>10 years	144	48.6
Employment role		
Full‐time	214	72.3
Part‐time	82	27.7
Overtime		
<1 h	191	67.7
≥2 h	91	32.3
Break in practice		
Yes	141	47.6
No	155	52.4
Employment type		
Permanent/ongoing	266	91.1
Fixed‐term contract	26	8.9
Type of service		
Public	186	63.1
Private	86	29.2
Both	23	7.8
Location		
Metropolitan	203	68.8
Rural	86	29.2
Both	6	2.0
State		
New South Wales	63	21.3
Victoria	63	21.3
Western Australia	60	20.3
Queensland	53	17.9
South Australia	28	9.5
ACT	16	5.4
Tasmania	9	3.0
Northern Territory	4	1.4
Career progression/advancement satisfaction		
Very satisfied	16	5.4
Satisfied	128	43.2
Neither satisfied nor dissatisfied	78	26.4
Dissatisfied	61	20.6
Very dissatisfied	13	4.4
Intention to change career		
Yes	28	9.5
Unsure	129	43.6
No	139	47.0
Intention to leave profession		
Yes	38	12.8
Unsure	85	28.7
No	173	58.4

### Associations and predictors of participating in research

Univariate analysis identified that additional qualifications and working in the public sector were more frequently associated with involvement in research, whereas, working in a metropolitan area and working in Western Australia (WA) was associated with less frequent research involvement. A significantly greater proportion of RTs who had or were currently completing additional qualifications had involvement in research projects (65%) compared to RTs who did not (39%) (X^2^ (1, *N* = 293) = 16.54, *P* < 0.001). A significantly smaller proportion of RTs who were located in metropolitan areas only (41%) had been involved in research projects compared to those located in rural/regional areas or both metropolitan and rural/regional areas (56%) (X^2^ (1, *N* = 295) = 6.24, *P* = 0.013). A significantly greater proportion of RTs employed by the public sector had been involved in research projects (57%) compared to those employed by the private sector (27%) or both the public and private sectors (26%) (X^2^ (2, *N* = 295) = 25.56, *P* < 0.001). A significantly smaller proportion of RTs who were working in WA had been involved in research projects (32%) compared to RTs who were working in other Australian states (50%) (X^2^ (1, *N* = 296) = 6.18, *P* < 0.013). No other significant differences existed between involvement in research projects and demographics or employment.

A regression model of personal and workplace characteristics found that working for a public service provider (OR = 3.2, 95% CI (1.7, 5.8), *P* < 0.001) and having additional qualifications (OR = 3.1, 95% CI (1.6, 6.0), *P* = 0.001) were relative predictors of RT involvement in research projects.

Only RTs who said they had been involved in research projects answered further questions about the details of their involvement in research.

### Research involvement

**Figure 1:**
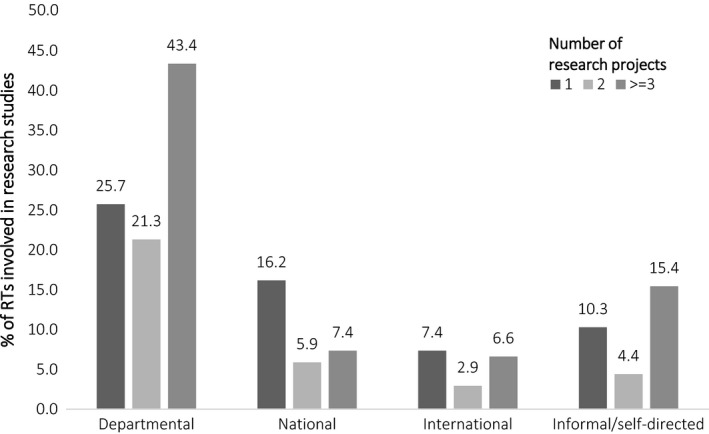
Number of departmental, national, international and informal/self‐directed research projects that RTs have been involved in.

Of the RTs involved in research studies, 91% (*n* = 124, 95% CI (87, 96)) had been involved in departmental, 28% (*n* = 38, 95% CI (20, 36)) in national, 14% (*n* = 19, 95% CI (8, 20)) in international, and 29% (*n* = 39, 95% CI (21, 36)) in informal or self‐directed research studies. Sixty‐six percent had been involved in at least 3 departmental projects, and 46% had been involved in at least three informal/self‐directed research projects. Sixteen percent of RTs had been part of one national research project and 7% had been involved in one international research project. There were fewer RTs who had been involved in more than one national (13%) research project (Fig. 1). Excluding informal/self‐directed projects, the median number of research projects RTs had been involved in was 3.0 (IQ 1.0–5.0). Treating the type of research as an ordinal outcome, there was a positive correlation between an RT's total number of research projects and the type of project involvement (departmental = 1, national = 2 and international = 3) with those participating in international projects also involved in a larger number of projects (*r*_*τ*_ = 0.449, *P* < 0.001).

### Sources of funding

Eleven RTs (8%, 95% CI (3, 13)) had been awarded research funding as a chief/principal investigator. Funding was received from: the Australian Institute of Radiography (now known as Australian Society of Medical Imaging and Radiation Therapy (ASMIRT)), Allied Health Training and Development scheme, state Cancer Councils (Victoria and New South Wales), Health Education and Training Institute, Frances and Harold Abbott Foundation, industry sponsored conference prize, Medical Radiation Technologists State Board, Victorian Cancer Agency and WA Radiation Oncology Small Grants Scheme.

### Research tasks

The main research tasks included: data collection (90%); data analysis (66%); literature review (60%); named investigator (45%); reporting and evaluating research (42%); ethics (34%); determination of research questions or hypotheses (33%); manuscript writing (33%); proposal development (31%); recruitment (25%); project manager (21%); grant application (16%) and other tasks (8%).

### Perspectives about being involved in research

Only two percent of RTs disliked conducting or being involved in research studies, with 35% describing involvement as ‘OK’, 47% as ‘liking it’ and a further 16% as ‘loving it’. Univariate tests revealed there were no significant differences between demographics or employment and RTs opinions of conducting research, satisfaction with career progression and advancement opportunities, intention to leave profession or intention to change career.

Approximately one‐third of RTs agreed/strongly agreed their involvement in research inspired them to stay in the profession (34%), 30% disagreed/strongly disagreed and 37% neither agreed nor disagreed. RTs without dependents were more likely to disagree/strongly disagree that involvement in research has inspired them to remain in their profession (35%) compared to RTs with dependents (10%) (X^2^ (2, *N* = 135) = 7.27, *P* = 0.026). A regression controlling for age confirmed that not having dependents was a relative significant predictor of disagreement with research inspiring RTs to remain in the profession. A significantly greater proportion of RTs who had/were currently completing additional qualifications agreed/strongly agreed that involvement in research has inspired them to remain in their profession (55%) compared to RTs without an additional qualification (22%) (X^2^ (2, *N* = 133) = 15.31, *P* < 0.001). Similarly, a significantly smaller proportion of RTs who had/were currently completing additional qualifications disagreed/strongly disagreed that involvement in research has inspired them to remain in their profession (18%) compared to RTs without an additional qualification (35%) (X^2^ (2, *N* = 133) = 15.31, *P* < 0.001). There were no other significant differences between RTs agreement that research inspired them to stay in the profession and demographics or employment, or between satisfaction with career progression and advancement opportunities, intention to leave profession or intention to change career.

### The impact of involvement in research projects on career perceptions

Participants provided responses to the following career perception items: satisfaction with career progression/advancement opportunities, intention to leave the profession and intention to change career (Table 2). Involvement in research projects was associated with a desire to make a career change. For example a significantly greater proportion of RTs who were involved in research projects (60%) were thinking of leaving their current workplace compared to those not involved in research projects (42%) (X^2^ (1, *N* = 296) = 5.95, *P* = 0.015). Similarly, a significantly greater proportion of RTs who had been involved in research intended to change roles, i.e. move into another position/role related to radiation oncology such as in an education or academic role (14%) compared to RTs who had not been involved in research projects (6%)(X^2^ [1, *N* = 296] = 5.98, *P* = 0.014). Comparably, a significantly greater proportion of RTs who had been involved in research projects were very satisfied with career progression/advancement opportunities (10%) compared with RTs who had not been involved in a research project (2%) (X^2^ (4, *N* = 296) = 10.85, *P* = 0.028). There were no other significant relationships between involvement in research projects and satisfaction with career progression and advancement opportunities, intention to leave profession or intention to change career.

A significantly greater proportion of RTs who intended to change roles, i.e. move into another position/role related to radiation oncology agreed/strongly agreed that their involvement in research had inspired them to remain in the radiation oncology profession (68%) compared with RTs who were unsure or did not intend to change roles (28%) (X^2^ (2, *N* = 135) = 11.66, *P* = 0.003). A significantly smaller proportion of RTs who intended to change roles neither agreed nor disagreed that their involvement in research had inspired them to remain in the radiation oncology profession (16%) compared with RTs who were unsure or did not intend to change roles (40%) (X^2^ (2, *N* = 135) = 11.66, *P* = 0.003).

### Research interests

Participants who had previous or desired involvement in research projects were asked to list the research areas in radiation oncology that interested them and a total of 159 respondents identified a broad number of areas with the most predominant including: treatment technique, patient focus and patient outcomes (Table 3). Outside of these topics, eight participants generally stated the need for conducting clinical trials and four suggested the need to conduct research that leads to evidence‐based practice.

**Table 3:** Research interests of RT respondents who had previous or desired research involvement (*n* = 159)

Topics	Sub topics^a^	Count (%)
Treatment technique	Treatment techniques	16 (10.1)
	Stereotactic radiotherapy (including SABR and SBRT)	10 (6.3)
	VMAT/IMRT	9 (5.7)
	Site‐Specific treatment techniques (e.g. lung, breast and prostate)	6 (3.8)
	Brachytherapy	2 (1.3)
	Head and neck adaptive radiotherapy	2 (1.3)
	Tomotherapy	2 (1.3)
	Gating	1 (0.6)
	Total body irradiation	1 (0.6)
Patient focus	Patient care	35 (22.0)
	Paediatric care	6 (3.8)
	Palliative care	1 (0.6)
	Rural patients	1 (0.6)
Patient outcomes	Patient outcomes and patient safety	15 (9.4)
	Side‐effect management (including skin care)	11 (6.9)
	Nutrition	3 (1.9)
	Radiobiology	1 (0.6)
	Treatment compliance	1 (0.6)
Technology	New technology	27 (17.0)
Imaging	Imaging and image‐guided radiation therapy (IGRT)	16 (10.1)
Workforce development and sustainability	Workforce Issues	8 (5.0)
	RT Education	4 (2.5)
	Advanced practice	2 (1.3)
	Occupational health and safety	1 (0.6)
Treatment planning	Planning	12 (7.5)
Treatment accuracy	Immobilisation	8 (5.0)
Department efficiency	Workflow	6 (3.8)
	Paperless department	1 (0.6)
	Patient management systems	1 (0.6)
Radiation safety	QA/QI and reducing errors	6 (3.8)
Complementary medicine	Complementary medicine	3 (1.9)
Multidisciplinary education	Education for multidisciplinary team/wider community of health professionals	1 (0.6)

a Multiple topics may be coded to a single response.

### Perceived benefits of conducting research

All participants were asked for their opinion of the perceived benefits of conducting research and 208 responded (Table 4). The benefits commonly identified by RTs included: develop new skills/increase knowledge; evidence‐based practice; keeping up to date and benchmarking and rewarding/challenging/job satisfaction.

**Table 4:** RT participants perceived benefits of conducting research (*n* = 208)

Benefits^a^	Count (%)
Develop new skills/increase knowledge	77 (37.0)
Evidence‐based practice, keeping up to date and benchmarking	60 (28.8)
Rewarding/challenging/job satisfaction	47 (22.6)
Benefit to community/patients/profession	36 (17.3)
Keeps them interested	22 (10.6)
Collaboration (MDT and within profession)	15 (7.2)
Career advancement	14 (6.7)
Recognition of work	7 (3.4)
Become an expert/teach others	5 (2.4)
Flexibility of research job and autonomy	2 (1.0)
Keep job	1 (0.5)

a Multiple benefits may be coded to a single response.

### Barriers to conducting research

Two hundred and six RTs provided their opinion of the barriers to conducting research. The perceived barriers of conducting research were predominantly linked to lack of time. Other barriers included lack of support, cost, lack of expertise and lack of incentive (Table 5).

**Table 5:** RT participants perceived barriers to conducting research (*n* = 206)

Barriers to conducting research^a^	Count (%)
Time and workload	152 (73.8)
Support	29 (14.1)
Funding	24 (11.7)
Education, expertise, confidence	24 (11.7)
Lack of motivation, incentive and interest	20 (9.7)
Career structure, lack of recognition and lack of pay for doing research	17 (8.3)
Family commitments	9 (4.4)
Lack of opportunities	9 (4.4)
Choosing a topic	7 (3.4)
Understaffed department	6 (2.9)
Part‐time	4 (1.9)
Politics, hierarchy and red tape	4 (1.9)
Ethics requirements	3 (1.5)
Self‐directed, repetitive and requires discipline	3 (1.5)
Lack of research culture	2 (1.0)
Access to data	2 (1.0)
Stress	2 (1.0)
Multidisciplinary links	2 (1.0)
Limited patient pool /patient participation	2 (1.0)

a Multiple barriers may be coded to a single response.

### Willingness for research project involvement

Forty‐one percent of RTs (*n* = 65, 95% CI (33, 48)) who had not previously been involved in research indicated that they wanted to be involved in research studies. A significantly greater proportion of RTs who had been working for 10 years or less (49%) wanted to be involved in research studies than those who had been working for greater than 10 years (31%) (X^2^ (1, *N* = 154) = 5.59, *P* = 0.018). No other significant differences existed between wanting to be involved in research projects and demographics or employment. A significantly smaller proportion of RTs who wanted to be involved in research did not think they would move into another position/role related to radiation oncology (e.g. at a tertiary institution in an education, academic or research role) (21%) compared to RTs who were unsure about changing roles (59%) or who intended to change roles (78%) (X^2^ (2, *N* = 160) = 28.71, *P* < 0.001). There were no other significant differences between RTs interest in being involved in research and satisfaction with career progression and advancement opportunities, intention to leave profession or intention to change career.

## Discussion

Almost half (46%) of the RT participants who responded to our workforce survey had been involved in some form of research. While not a direct comparison, Wright, Hilder and Schenider‐Kolsky reported that 36% of clinical centres in Australia in 2009 had research RTs (including full‐time or part‐time roles and some integrated with clinical or education roles); however, they did not report on individual RTs involvement in research.^2^ In this study, RTs may have been employed as research RTs, participated in research in their clinical roles, or as university‐based academics. Working in the public sector, in rural/regional areas and having additional qualifications were significantly associated with being involved in research.

Twenty percent of RT participants had post‐graduate qualifications; however, some may have completed a Graduate Entry Master by Coursework which is a profession entry qualification rather than intensive research training. Five participants reported that they had research‐based post‐graduate qualifications. In comparison, Wright, Hilder and Schneider‐Kolsky reported that 32 RTs were completing or had completed Higher Degrees by Research.^2^ More recently Ekpo et al.^21^ reported that 15 RTs had completed doctoral studies in Australia. The total number of Australian RTs with Higher Degrees by Research is not published, but it is likely this study did not capture all RTs who have completed or enrolled in Higher Degrees by Research or those who are participating in research. However, it did capture a proportion of RTs who were participating in research and their perspectives. Furthermore, given the total number of RT participants, the percentage of RTs with post‐graduate qualifications in this study is likely to be representative of RTs in Australia with post‐graduate qualifications.

Involvement in research projects was associated with intention to leave their workplace or change roles. This may be because RTs wanted to increase their involvement in research or further their qualifications. If more opportunities to participate in research were available RTs may be less inclined to leave their workplace. Having the opportunity to be involved in research inspired RTs to remain in the profession, while expanding their roles or moving to an education or academic role. Also an interest in conducting research amongst RTs who had no experience was associated with less likelihood of changing roles.

Less than 10% of RT participants who had participated in research had led a project and received funding as a chief investigator. Participants reported that they had received funding from State‐based opportunities as well as professional bodies. In comparison, Wright, Hilder and Schneider‐Kolsky reported that radiation oncology centres also received funding from the NHMRC.^2^ Interestingly, participants in this study had not received funding from the NHMRC; however, we are aware that NHMRC and Cancer Australia funded trials are being run in radiation oncology centres. This suggests that these projects are often not being led by RTs and that there are opportunities for RTs to develop their research skills and lead projects relevant to radiation therapy practice. However, it is also necessary to acknowledge that a small number of RTs leading NHMRC/Cancer Australia funded projects may not have responded to this survey. Project initiation/conceptualisation‐related tasks were reported by RTs less often as was manuscript writing. This may suggest the need to provide RTs with further research education and opportunities to participate in developing projects and publishing results.

A wide range of research topics were identified by RTs including treatment and technique, patient care and outcomes, workforce and department efficiency. This finding extends previous research.^7^, ^8^, ^9^, ^10^ Forty percent of RTs with no research experience were interested in research. A desire to be involved in research was associated with working for less than 10 years and no intention to change roles. A younger workforce with research ambitions is a positive opportunity for the profession, particularly in comparison to trends seen in other areas of health research such as primary care.^22^, ^23^ This may represent an opportunity for the radiation therapy profession to increase research activity and further develop a research culture. RTs may benefit from support and mentorship, time and more opportunities to become involved in research.^3^, ^4^, ^5^ Ward et al.^24^ recently summarised the importance of research mentorship and provided guidance on how mentorship can be provided in the clinical setting.

Similar to our work with ROMPs^12^ and our previous qualitative work^11^ this study found that RTs enjoyed involvement in research and perceived research participation was beneficial for professional development and participating in best practice. Furthermore, participation in research was linked with job satisfaction. This finding has also been reported for radiation oncologists.^25^ The barriers of conducting research were predominantly linked to lack of time and current workload. This finding in radiation therapy is not new^3^, ^4^, ^5^ and similar barriers to conducting research in the oncology setting are reported elsewhere.^26^ Also highlighted in this study was the need for support and expertise, which has similarly been reported elsewhere.^3^, ^4^, ^5^

We have demonstrated that RTs are keen to participate in research that improves evidence‐based practice and their involvement has increased over time. Furthermore, some RTs are now taking lead roles in conducting research, which has been reported elsewhere.^27^ Previous research in radiation oncology has highlighted that research capacity is often displaced by routine clinical duties because the benefits of being involved in research have not been adequately recognised.^6^, ^11^, ^28^, ^29^ However, with more RTs participating in research and volunteering their skills it may be possible to change this attitude to research and advocate for more opportunities and time allocated to research. In addition, RTs will need to be proactive in their strategies to obtain research funding (e.g. obtaining mentors; applying for new investigator grants; publishing in high impact journals and collaborating strategically) to facilitate their leadership of research projects.

With more RTs involved in research it will be possible for RTs to support each other as well as seeking support from the multidisciplinary team. A research network known as the Australian and New Zealand Medical Radiations Research Network (www.anzmrrn.org) has been established to facilitate collaboration and enable medical radiation practitioners to support each other. Gillan et al.^27^ provide ideas for building a research culture for RTs and highlight the need for knowledge sharing between RTs. Furthermore, Rosewall et al.^30^ demonstrated that in their radiation therapy centre in Canada they were able build research capacity by introducing research education, establishing a research committee, implementing research RT positions, informing staff about research publications and success and holding networking opportunities. This was subsequently followed by an article demonstrating that these research activities were sustained and increased over a 10 years period.^5^ In WA we have established a radiation oncology workforce WA group and aim to: hold research education sessions and meetings for professionals to form initial collaboration and discuss ideas; establish additional topic‐based research support groups and provide opportunities for researchers to receive feedback on their work; provide incentives/recognition for radiation oncology professionals involved in research; and implement a database of ongoing and completed research projects, published papers and current research opportunities.

### Limitations

Two‐hundred and ninety‐six RTs participated in this study, representing 13% of RTs registered in Australia in 2014 when the data were collected.^31^ In comparison to RTs registered nationally, the distribution of participants by states was approximately similar in this study, apart from a greater proportion of participants in WA and a smaller proportion in New South Wales.^31^ This study provides an understanding of Australian RTs' perspectives towards research. RTs volunteered to participate in the larger workforce survey, and the potential for selection bias needs to be acknowledged because their workforce opinions may have also impacted on their engagement with research and their decision to participate. For example RTs with strong positive or negative opinions of working in their profession or RTs who had time to respond when the survey was distributed may have been more likely to respond. While this sample is a small proportion of RTs in Australia and may not have been representative of all research active RTs in Australia it does provide us with an understanding of RTs' perspectives, research topic areas and perceived benefits and barriers. Furthermore, it demonstrates that although the number of RTs participating in research has increased, there is still a need to continue to encourage and support RT involvement in research.

## Conclusion

Almost half of the RTs who participated in this study identified that they were participating in research. However, the degree and amount of participation varied. More time and support needs to be provided to RTs to enable them to actively participate in research and build research capacity in radiation therapy. We demonstrated RTs have identified many research topics that would improve evidence‐based practice and the quality of care provided to patients. Furthermore, continued involvement and opportunities in research are likely to improve RTs job satisfaction. Future research should focus on ongoing measurement of RT research involvement, developing methods to support RTs conducting research and building research capacity.

## Conflict of Interest

The authors declare no conflict of interest.

## References

[1] Gambling T , Brown P , Hogg P . Research in our practice – a requirement not an option: Discussion paper. Radiography 2003; 9: 71–6.

[2] Wright C , Hilder B , Schneider‐Kolsky M . Meeting the research agenda in Australia radiation therapy: The current picture. J Radiother Pract 2009; 8: 67–77.

[3] Scutter S , Halkett G . Research attitudes and experiences of radiation therapist. Radiographers 2003; 50: 69–72.

[4] Turner A , D'Alimonte L , Fitch M . Promoting radiation therapy research: Understanding perspectives, transforming culture. J Radiother Pract 2013; 12: 92–9.

[5] Higgins J , Davey C , Li W , Chan K , Wenz J , Rosewall T . 10 years of exposure to a radiation therapist research culture: Where are we now? J Med Imaging Radiat Sci 2011; 42: 106–12. 10.1016/j.jmir.2011.04.004 31051856

[6] Agustin C , Grand M , Gebski V , Turner S . Radiation therapists' perspective on barriers to clinical trials research. J Med Imaging Radiat Oncol 2008; 52: 178–82. 18373811 10.1111/j.1440-1673.2008.01938.x

[7] Halkett GK , Cox J , Anderson C , Heard R . Establishing research priorities for Australian radiation therapists: What patient care priorities need to be addressed? Eur J Cancer Care 2012; 21: 31–40. 10.1111/j.1365-2354.2011.01276.x 21838723

[8] Cox J , Halkett G , Anderson C , Heard R . A Delphi Study on research priorities in radiation therapy: The Australian perspective. Radiography 2010; 16: 26–39.

[9] Cox J , Halkett G , Anderson C . Research interests identified at the coal‐face: Initial Delphi analysis of Australian radiation therapists' perspectives. Radiographers 2009; 56: 9–14.

[10] Egestad H , Halkett G . A Delphi study on research priorities in radiation therapy: The Norwegian perspective. Radiography 2016; 22: 65–70.

[11] Halkett G , McKay J , Hegney D , et al. Radiation Therapists' and Radiation Oncology Medical Physicists' perceptions of work and the working environment in Australia. Eur J Cancer Care 2016; https://doi.org/10.1111/ecc.12511. 10.1111/ecc.12511 27147506

[12] Ebert MA , Halkett GK , Berg M , et al. An assessment of radiation oncology medical physicists' perspectives on undertaking research. Australas Phys Eng Sci Med 2017; 40: 173–80. 27900621 10.1007/s13246-016-0505-3

[13] Hegney D , Eley R , Plank A , Buikstra E , Parker V . Workforce issues in nursing in Queensland: 2001 and 2004. J Clin Nurs 2006; 15: 1521–30. 17118074 10.1111/j.1365-2702.2006.01558.x

[14] Department of Health and Ageing . Radiation Oncology Workforce Planning Final Report. Sydney, Australia, HealthConsult Pty Ltd, 2009.

[15] Hegney D , Tuckett A , Parker D , Robert E . Access to and support for continuing professional education amongst Queensland nurses: 2004 and 2007. Nurse Educ Today 2010; 30: 142–9. 19646799 10.1016/j.nedt.2009.06.015

[16] Imle M , Atwood J . Retaining qualitative validity while gaining quantitative reliability and validity: Development of the Transition to Parenthood Concerns Scale. ANS Adv Nurs Sci 1988; 11: 61–75. 3140722 10.1097/00012272-198810000-00007

[17] Monterosso L , Kristjanson L , Dadd G . Content validity and reliability testing of the FIN‐PED II: A tool to measure care needs of parents of children with cancer. J Nurs Meas 2006; 14: 31–44. 16764176 10.1891/jnum.14.1.31

[18] Lynn MR . Determination and quantification of content validity. Nurs Res 1986; 35: 382–5. 3640358

[19] Vaismoradi M , Turunen H , Bondas T . Content analysis and thematic analysis: Implications for conducting a qualitative descriptive study. Nurs Health Sci 2013; 15: 398–405. 23480423 10.1111/nhs.12048

[20] Hsieh H , Shannon SE . Three approaches to qualitative content analysis. Qual Health Res 2005; 15: 1277–88. 16204405 10.1177/1049732305276687

[21] Ekpo EU , Snaith B , Harris MA , McEntee MF . Doctoral profile of the medical radiation sciences: A baseline for Australia and New Zealand. J Med Radiat Sci 2017; https://doi.org/10.1002/jmrs.231. 10.1002/jmrs.231 PMC5587660 28440052

[22] Winzenberg TM , Gill GF . Prioritising general practice research. Med J Aust 2016; 205: 55–7. 27456440 10.5694/mja16.00578

[23] Anderson W . NHMRC funding for primary care research 2000–2008. Med J Aust 2011; 195: 583. 22107004 10.5694/mja11.11252

[24] Ward E , Hargrave C , Brown E , Halkett G , Hogg P . Achieving success in clinically based research: The importance of mentoring. J Med Radiat Sci 2017; https://doi.org/10.1002/jmrs.234. 10.1002/jmrs.234 PMC5715317 28653426

[25] Leung J , Rioseco P , Munro P . Stress, satisfaction and burnout amongst Australian and New Zealand radiation oncologists. J Med Imaging Radiat Oncol 2015; 59: 115–24. 25088562 10.1111/1754-9485.12217

[26] Johnson C , Lizama C , Harrison M , Bayly E , Bowyer J , Haddow L . Cancer health professionals need funding, time, research knowledge and skills to be involved in health services research. J Cancer Educ 2014; 29: 389–94. 24510803 10.1007/s13187-014-0625-y

[27] Gillan C , DiProspero L, Harnett N, Holden L (eds). Research for the Radiation Therapist: From Question to Culture. Apple Academic Press Inc, Oakville, Canada, 2014.

[28] Mackay RI , Burnet NG , Green S , Illidge TM , Staffurth JN ; on behalf of the NCEG . Radiotherapy physics research in the UK: Challenges and proposed solutions. Br J Radiol 2012; 85: 1354–62. 22972972 10.1259/bjr/61530686 PMC3474027

[29] Sehlen S , Vordermark D , Schäfer C , et al. Job stress and job satisfaction of physicians, radiographers, nurses and physicists working in radiotherapy: A multicenter analysis by the DEGRO Quality of Life Work Group. Radiat Oncol 2009; 4: 6. 19200364 10.1186/1748-717X-4-6 PMC2661891

[30] Rosewall T , Kelly V , Higgins J , et al. The influence of programmatic change on radiation therapist research capacity – A single‐center case study. J Med Imaging Radiat Sci 2009; 40: 170–7. 10.1016/j.jmir.2009.08.002 31051828

[31] Australian Health Practitioner Regulation Agency . Annual Report 2013/14: The Australian Health Practitioner Regulation Agency and the National Boards, reporting on the National Registration and Accreditation Scheme. Australian Health Practitioner Regulation Agency, Melbourne, Australia, 2014.

